# Understanding Mental Burden and Factors Associated With Study Worries Among Undergraduate Medical Students During the COVID-19 Pandemic

**DOI:** 10.3389/fpsyg.2021.734264

**Published:** 2021-12-15

**Authors:** Jennifer Guse, Ines Heinen, Sonja Mohr, Corinna Bergelt

**Affiliations:** ^1^Department of Medical Psychology, University Medical Center Hamburg-Eppendorf, Hamburg, Germany; ^2^Dean’s Office for Student Affairs, Faculty of Medicine, University Medical Center Hamburg-Eppendorf, Hamburg, Germany; ^3^Department of Medical Psychology, University Medicine Greifswald, Greifswald, Germany

**Keywords:** mental health, medical education, COVID-19, learning environment, undergraduate medical students

## Abstract

The Coronavirus disease 2019 (COVID-19) pandemic is affecting many areas of life and has led to major changes in undergraduate medical education. Even before the COVID-19 pandemic, high mental burden of medical students has frequently been reported in the literature. Additional pandemic-specific stressors could exacerbate this situation. This study aimed to assess mental health outcomes among medical students during the first semester after the COVID-19 outbreak and perception of the students on how the learning environment has changed. In May 2020, we conducted a cross-sectional survey among undergraduate medical students at a large medical school in Germany. The survey included validated mental health instruments (Distress Thermometer, Patient Health Questionnaire 4) and self-developed items to examine the perception of the study situation during the COVID-19 pandemic. Open-ended questions were analyzed by conventional content analyses. The response rate was 59.2% (914/1,545). Overall, 61.9% of the students reported distress levels above the cutoff. Year 1 students reported significantly higher levels of distress, anxiety and depression than students during their second to fourth year of studies. 48.3% of the students indicated a decrease in their study motivation since the beginning of the COVID-19 pandemic with significant differences between study years. The binary logistic regression model showed that male gender, being in study year 2, higher distress scores and higher symptoms of depression were significantly associated with a higher likelihood for experiencing serious worries. In the open-ended questions on current concerns related to the impact of the COVID-19 pandemic on their studies, students most frequently reported concerns about missing relevant practical learning experience, difficulties with self-regulated learning and self motivation as well as study-related worries. Year 4 students reported significantly more worries about the lack of practical training than students from study years 1 to 3. Analysis of gender differences showed that female students reported more frequently diverse worries. In contrast, female students shared more frequently helpful strategies in all the categories compared to male students. Our findings suggest that medical students experience significant levels of distress and mental burden during the COVID-19 pandemic and highlight the need for ongoing psychological and educational support for medical students during the COVID-19 pandemic and after.

## Introduction

Since December 2019, reports of an illness with a novel coronavirus disease 2019 (COVID-19) had been accumulating from the Wuhan region of China and infections had been multiplying at a rapid rate worldwide (Zhu et al., 2020), prompting the World Health Organization (2020a) to declare an international the COVID-19 pandemic. The COVID-19 pandemic has been affecting many areas of life. Due to the rapid increase in the number of COVID-19 infections, many governments around the world have imposed strict rules on domestic quarantine and social isolation.

On January 27, 2020, the German Federal Ministry of Health (2020) announced the first COVID-19 case had been detected in Germany. Since then, the epidemiological situation has deteriorated sharply. As of May 31, 2020, more than 180,000 people in Germany had already been infected with COVID-19, of which 8,500 cases (4.7% of all the confirmed cases) have been fatal (Robert-Koch-Institut, 2020). At that time, Hamburg was the third most affected German state in terms of population (cases/100,000 inhabitants) (Robert-Koch-Institut, 2020). Worldwide, there were nearly 6 million confirmed cases at that time, including 367,166 deaths, according to the World Health Organization (2020b). At this time, the COVID-19 pandemic was spreading most rapidly in North and South America. The United States and Brazil were affected the most, with more than 100,000 new infections within 7 days (May 25–31, 2020). Most new infections were measured in Germany from mid-March to around mid-April 2020 (Robert-Koch-Institut, 2020). The rapid increase in the number of infections noticeably restricted the everyday life of the population. Since mid-March 2020, the German government announced several restrictions with respect to public life to suppress the spread of COVID-19 by increasing social distancing, i.e., school, daycare, and nonessential shop closures, bans on public meetings (Steinmetz et al., 2020). Depending on the federal state in Germany, people were not allowed to meet more than one person from another household, and schools, daycare and nonessential shops were closed. Temporarily people were not allowed to leave their homes without a reason (Steinmetz et al., 2020). At universities, lectures and seminars have been held predominantly digitally to reduce interpersonal contact and protect patients, students, and faculty (HRK German Rectors’ Conference, 2020). Consequently, the spread of the COVID-19 pandemic has caused major changes in undergraduate medical education, too (Whelan et al., 2020). Many medical schools disrupted their undergraduate medical education and transitioned most of their teaching to digital formats (Rose, 2020). In Germany, it was agreed in March 2020 that medical lectures were held predominantly in digital form until further notice (Deutsche Hochschulmedizin, 2020). In this context, the learning environment as well as the study and examination conditions for students changed significantly.

A recent conceptual framework proposed a learning environment that encompasses a psychosocial dimension with a personal, social, and organizational component in addition to a material dimension that includes physical and virtual spaces (Gruppen et al., 2019). According to Gruppen et al. (2019), these five core components overlap and interact with each other. The personal and social components strongly shape perceptions of the learning environment of the students. Furthermore, the personal component reflects the psychological characteristics of the learners, e.g., quality of life, moral distress, and worries about future endurance. The social component describes the quality of interactions between peers, students and faculty, and students and patients (Gruppen et al., 2019). These interactions of learners include, for example, cooperation and competition with peers, feedback by and communication with faculty, and responsibility for and acceptance by the patients. In light of the COVID-19 pandemic, the personal and social components, in particular, could be seriously compromised. Previous studies highlighted the impact of the learning environment on well-being of the medical students (Dyrbye et al., 2009, 2020). Learning environments that are perceived as unsupportive and less nurturing are typically associated with decreased mental health among medical students (Dyrbye et al., 2009; Schwenk et al., 2010; Wasson et al., 2016). An international study has explored the impact of favorable perceptions of the learning environment (LE) on self-reported quality of life, burnout, and empathy of the undergraduate students at three different medical schools (Tackett et al., 2017). In total, 62% of the sample reported more favorable than unfavorable LE perceptions, which were positively correlated with better quality of life, lower emotional exhaustion, and less depersonalization in adjusted models. The domain “community of peers” as one factor of the applied instrument was the only factor that was independently associated with better quality of life scores, less emotional exhaustion, and less depersonalization (Tackett et al., 2017). A recent systematic review explored the association between learning environment interventions and improved mental health among undergraduate medical students (Wasson et al., 2016). The study group identified 28 of 4,207 published articles including more than 8,000 participants that met the inclusion criteria of their review. The results indicated that pass/fail grading systems (compared to grading systems with three or more intervals), formal mentoring/advisor programs, mental health and wellness programs were associated with improved emotional well-being among medical students.

Even before the COVID-19 pandemic, high mental burden of medical students had frequently been reported in the literature (Dyrbye et al., 2006; Hope and Henderson, 2014; Heinen et al., 2017). Additional pandemic-specific stressors could exacerbate this situation. In a study conducted in March 2020, 25% of the college students at a medical school in China reported anxiety related to COVID-19 (Cao et al., 2020). Previous research had reported the negative impact of past pandemics (Hawryluck et al., 2004) and the COVID-19 pandemic (Wang et al., 2020) on the general population and on specific groups, e.g., medical students (Elmer et al., 2020) and health professionals (Lai et al., 2020). Health professionals may be particularly affected (Bao et al., 2020) because of additional stressors on top of the general pandemic-specific ones (Inter-Agency Standing Committee (IASC), 2020). A number of recent studies have shown that mental burden during the COVID-19 pandemic was highest among women and young adults (Elmer et al., 2020; Pieh et al., 2020; Dale et al., 2021). For example, an Austrian study investigated mental health in the Austrian population during a strict lockdown in December 2020/January 2021 and found a prevalence of 26% for moderate depression, 23% for moderate anxiety, and 18% for moderate insomnia (Dale et al., 2021). For all the measures, women reported a higher mental burden than men. Likewise, the youngest age group (18–24 years) reported statistically significant more mental health symptoms in comparison to the oldest age group (65+ years) (Dale et al., 2021).

This study aimed to assess mental health outcomes among medical students during COVID-19 and perception of the students with respect to how learning environment has changed in a large sample of undergraduate medical students in Germany. It was conducted with the purpose of better understanding their levels of distress, anxiety, and depression as well as their perception of the learning environment during the first semester after the COVID-19 outbreak. This study is exploratory in nature. We investigated demographic and mental health factors associated with serious worries about the study situation during the COVID-19 pandemic and addressed the following two research questions: (1) Do female and male students differ with regard to mental burden and study worries during the COVID-19 pandemic? and (2) Do students in different years of study differ in terms of psychological distress and study worries during the COVID-19 pandemic? Based on recent studies (Elmer et al., 2020; Pieh et al., 2020; Dale et al., 2021), it is expected that female students might be higher burdened in contrast to male students, and students in the first study years might be higher burdened than students in year 2–4 as age increases with study year.

## Materials and Methods

### Study Design

We conducted a cross-sectional online survey at the University Medical Center Hamburg-Eppendorf to measure mental health and perception of the learning situation of the medical students during and after the initial phase of the COVID-19 pandemic. The anonymous survey started 6 weeks after the summer term started on May 28, 2020 and ended on June 7, 2020.

### Participants

All medical students (*n* = 1,545) enrolled in the integrated medical degree program (iMED) at the Medical Faculty of the University of Hamburg (Rheingans et al., 2019) in the summer semester 2020 were invited to participate in the online survey. Students were asked to complete an anonymous questionnaire linked to the voluntary curriculum evaluation conducted by the Dean’s Office at regular intervals throughout the year. A few days in advance, students were informed by email about the study objectives, voluntary participation, and privacy policies. Out of 1,545 students, 887 students completed the questionnaire in full, of which 63.4% were women. Respondents were spread across study years 1 to 4 of the undergraduate medical curriculum (*n* = 307, 192, 210, and 178 for years 1–4, respectively). Most of the students were between 21 and 25 years old. Details are shown in Table 1.

**TABLE 1:** Sample characteristics (*n* = 887).

	Whole sample		Study year			
			Year 1 (*n* = 307)	Year 2 (*n* = 192)	Year 3 (*n* = 210)	Year 4 (*n* = 178)
	*n*	%	%	%	%	%
**Sex**						
Female	562	63.4	59.9	64.6	61.4	59.9
Male	325	36.6	40.1	35.4	38.6	40.1
**Age**						
≤ 20 years	199	22.4	44.6	28.6	3.3	44.6
21–25 years	461	52.0	31.9	50.0	69.5	31.9
≥ 26 years	227	25.6	23.5	21.4	27.1	23.5

*n: frequencies.*

### Outcomes/Measures

We used the ultra-brief version of established measures of mental health that were validated in German and that have been used in previous studies (Heinen et al., 2017) to increase comparability. Longer versions of the instruments were used in recent studies during the COVID-19 pandemic in representative samples (Lai et al., 2020; Dale et al., 2021) and among students (Elmer et al., 2020). Additionally, we developed tailored items to assess perceptions of the students.

#### Distress

We used the German version of the Distress Thermometer (DT). The DT is a reliable and efficient single-item screening instrument with a scale from 0 to 10 developed by the National Comprehensive Cancer Network (Mehnert et al., 2006). Higher scores indicate higher distress. Internationally, a cutoff score of 5 and higher is established as a signal that a person is distressed and needs support.

#### Depression and Anxiety

We examined depression and anxiety with the German version of the four-item Patient Health Questionnaire-4 (PHQ-4). The ultra-brief screening instrument consists of a two-item depression scale (PHQ-2) and a two-item anxiety scale [Generalized Anxiety Disorder-2 (GAD-2)] and measures the amount of depression and anxiety symptoms the individual has felt during the past 2 weeks (Kroenke et al., 2009). The PHQ-4 total score is an overall measure of symptom burden using the following categories: 0–2 (normal), 3–5 (mild), 6–8 (moderate), and 9–12 (severe). It is a reliable screening instrument with good psychometric properties among students (Khubchandani et al., 2016).

#### Perception of Study Situation During Coronavirus Disease 2019 Pandemic

To measure direct changes in the study motivation and perceptions of the educational situation in the context of digital teaching, we employed two self-developed items: (1) “Has your study motivation changed since the start of the COVID-19 pandemic?” with three options to answer (increased, unchanged, and decreased) and (2) “Has your assessment of your study situation (e.g., teaching and learning conditions, scheduling, graduation opportunities, etc.) changed in the context of the COVID-19 pandemic?” with three answer options (No, I am as worried or unworried as before; Yes, I am somewhat worried; and Yes, I am seriously worried). Furthermore, students were asked for free-text answers to the questions “What comes to mind first when thinking about your current study situation?” and “What comes to mind as particularly helpful in your current situation?”.

### Statistical Analysis

#### Quantitative Data

The primary analysis involved descriptive statistics (numbers, percentages, means, and SDs) for demographic data and for estimating the magnitude of distress, the degree of symptoms of anxiety and depression, the prevalence of serious worries with respect to the current educational situation and the perception of the current educational situation of the students. Group comparisons were carried out using the chi-squared test for categorical variables and the *t*-test or ANOVA for differences of means. A two-tailed *p* < 0.05 was considered as statistically significant. Descriptive analyses were conducted to examine the magnitude of distress (DT), depression and anxiety (PHQ-4). The results of the entire sample were compared with the German norm population (Löwe et al., 2010; *n* = 5,030, mean age = 48 years, 54% women) and with PHQ-4 data of a German medical student sample (*n* = 321, mean age = 22 years, 60% women) from a previous study at the same faculty (Heinen et al., 2017) with the one-sample *t*-test. The binary logistic regression model was conducted to identify associations of the independent variables with serious worries (dichotomous) with respect to the study situation during the COVID-19 pandemic. Therefore, the sample was divided into two groups according to reported worries of the students (not or somewhat worried vs. seriously worried). We used forward and backward stepwise procedures to confirm that the results were stable and generalizable, independent of the model approach used. The independent variables included: sex, age (in groups), year in medical school, distress, anxiety, and depression. Nonsignificant variables were excluded stepwise via forward elimination and dropped at the level of *p* < 0.05. To avoid multicollinearity, we analyzed variance inflation factors (VIFs) scores (Midi et al., 2013). All the quantitative analyses were carried out using IBM SPSS software version 27 (SPSS software, IBM Corporation, New York, United States).

#### Qualitative Data

To analyze the qualitative data obtained by the open-ended questions, we conducted conventional content analyses with inductive categorization (Hsieh and Shannon, 2005). Two researchers (IH and JG) independently identified key concepts and created coding labels for recurring themes. Next, both the independently sorted codes into categories were reviewed by all the authors. Final definitions for categories and codes were developed by consensus and examples of each category were selected for illustration and translated into English. All the qualitative analyses were carried out using MAXQDA 2020 (VERBI Software, 2019).

After inductive categorization, responses of the students for each category were dichotomized (mentioned vs. not mentioned) to increase data transparency and to support our interpretation (Monrouxe and Rees, 2020). When students indicated more than one category per response, all responses were categorized. Group comparisons were conducted using the chi-squared test.

#### Ethical Considerations

The local ethics board of the Center for Psychosocial Medicine at the University Medical Center Hamburg-Eppendorf approved this study (LPEK-0161).

## Results

### Quantitative Data

The response rate was 59.2% (914/1,545). Responses of 27 students had to be excluded due to missing data in age or gender. Thus, the final sample included 887 students for analyses (63.4% females). The majority was aged 21 to 25 years. The demographic characteristics of the final sample are shown in Table 1.

Results showed that 61.9% of the students reported distress levels above the recommended cutoff score. The level of distress (as measured by the DT) as well as anxiety and depression (as measured with the PHQ-4) differed statistically significant for the different study years. Year 1 students reported the highest mean scores (Table 2).

**TABLE 2:** Number of students above and below the cutoffs and mean scores for distress, depression and anxiety, perception of the study situation during the COVID-19 pandemic, and changes of study motivation for the total sample and by study year.

		Total	Study year				
		(*n* = 887)	Year 1 (*n* = 307)	Year 2 (*n* = 192)	Year 3 (*n* = 210)	Year 4 (*n* = 178)	Statistic
**DT score**	< 4	338 (38.1)	52 (16.9)	95 (49.5)	108 (51.4)	83 (46.6)	χ^2^ (3) = 90.14,
*n* (%)	≥ 5	549 (61.9)	255 (83.1)	97 (50.5)	102 (48.6)	95 (53.4)	***p* < 0.001**
**PHQ-2 score**	< 3	704 (79.4)	220 (71.7)	153 (79.7)	189 (90.0)	142 (79.8)	χ^2^ (3) = 25.66,
*n* (%)	≥ 3	183 (20.6)	87 (28.3)	39 (20.3)	21 (10.0)	36 (20.2)	***p* < 0.001**
**GAD-2 score**	< 3	723 (81.5)	219 (71.3)	165 (85.9)	189 (90.0)	150 (84.3)	χ^2^ (3) = 34.53,
*n* (%)	≥ 3	164 (18.5)	88 (28.7)	27 (14.1)	21 (10.0)	28 (15.7)	***p* < 0.001**
**DT score**							Welch’s *F*(3, 453.03) = 47.76,
*M (SD)*		5.17 (2.53)	6.46 (2.31)	4.48 (2.26)	4.41 (2.50)	4.58 (2.35)	***p* < 0.001**,
**PHQ-4 score**		3.13 (2.46)	3.95 (2.57)	2.81. (2.29)	2.40 (2.02)	2.93 (2.53)	Welch’s *F*(3, 457.94) = 20.54,
*M (SD)*							***p* < 0.001**
**Worries about study situation**							χ^2^(6, *N* = 855) = 22.23,
Not worried	*n* (%)	293 (34.3)	115 (39.8)	45 (23.7)	82 (40.8)	51 (29.1)	***p* = 0.001**
Somewhat worried	*n* (%)	458 (53.6)	149 (51.6)	114 (60.0)	96 (47.8)	99 (56.6)	
Seriously worried	*n* (%)	104 (12.2)	25 (8.7)	31 (16.3)	23 (11.4)	25 (14.3)	
**Changes of study motivation**							χ^2^ (6, *N* = 855) = 25.57,
Increased	*n* (%)	116 (13.1)	41 (13.4)	17 (8.9)	36 (17.1)	22 (12.4)	***p* < 0.001**
Unchanged	*n* (%)	343 (38.7)	140 (45.6)	57 (29.7)	71 (33.8)	75 (42.1)	
Decreased	*n* (%)	428 (48.3)	126 (41.0)	118 (61.5)	103 (49.0)	81 (45.5)	

*DT, Distress Thermometer (range 0–10); n, frequencies; χ^2^, chi-squared; p, p-value; PHQ-2, Patient Health Questionnaire-2 (range 0–6); GAD-2, Generalized Anxiety Disorder-2 (range 0–6); M, mean; PHQ-4, Patient Health Questionnaire-4 (range 0–12); COVID-19, coronavirus disease 2019.*

*Bold font indicates statistical significance.*

Compared to a German norm population (Löwe et al., 2010; PHQ-4: *M* = 1.76; *SD* = 2.06), the medical students in this study reported significantly higher levels of anxiety and depression (PHQ-4: *M* = 3.13, *SD* = 2.46, *p* < 0.001) and in comparison to the group of medical students from a prior study at the same medical school (Heinen et al., 2017; *M* = 2.65, *SD* = 2.20, *p* < 0.001). Details are shown in Table 3.

**TABLE 3:** Mean scores for depression and anxiety among medical students during the COVID-19 pandemic in May 2020 for the total sample in comparison to the German norm population (Löwe et al., 2010) and medical students at the same medical school in 2014 (Heinen et al., 2017).

		Medical students 2020 during the COVID-pandemic	German norm population (Löwe et al., 2010)	p	d
		*n* = 887	*n* = 5,030		
PHQ-4 score	M (SD)	3.13 (2.46)	1.76 (2.06)	** < 0.001**	0.645
PHQ-2 score	M (SD)	1.70 (1.35)	0.94 (1.20)	** < 0.001**	0.621
GAD-2 score	M (SD)	1.43 (1.43)	0.82 (1.10)	** < 0.001**	0.528

		**Medical students 2020 during the COVID-pandemic**	**Medical students 2014 (Heinen et al., 2017)**	**p**	**d**
		***n* = 887**	***n* = 321**		

PHQ-4 score	M (SD)	3.13 (2.46)	2.65 (2.20)	** < 0.001**	0.201
PHQ-2 score	M (SD)	1.70 (1.35)	1.26 (1.12)	** < 0.001**	0.340
GAD-2 score	M (SD)	1.43 (1.43)	1.40 (1.36)	0.587	0.021

*PHQ-4, Patient Health Questionnaire-4 (range 0–12); M, mean; n, frequencies; p, p-value; d, effect size Cohen’s d; PHQ-2, Patient Health Questionnaire-2 (range 0–6); GAD-2, Generalized Anxiety Disorder-2 (range 0–6).*

*According to Cohen’s (1988) guidelines, we considered d = 0.2 to be a small effect, d = 0.5 as a medium effect, and d = 0.8 as a large effect.*

*Bold font indicates statistical significance.*

Analysis of gender differences showed that relatively more male students reported depression levels (PHQ-2) above the established cutoff than female students [male: 24.3% vs. female: 18.5%; χ^2^ (1, *n* = 887) = 4.23, *p* = 0.04]. Nevertheless, data revealed no further significant differences between male and female students with respect to the self-reported level of DT and overall anxiety and depression (PHQ-4).

Overall, 48.3% of medical students reported a decrease in their study motivation since the beginning of the COVID-19 pandemic with significant differences between study years (*p* < 0.001). Relatively, the proportion of students reporting a decreased motivation was highest among year 2 students (Table 2). A significantly higher proportion of male students (54.2%) suffered from a decreased study motivation than female students [44.8%, χ^2^ (2, *n* = 887) = 9.36, *p* = 0.01].

The majority of students was somewhat worried about the impact of the COVID-19 pandemic on their study situation, 34.3% reported to be as worried as before and 12.2% reported to be seriously worried (Table 2). Again, relatively more male students were burdened with serious worries than female students [15.7 vs. 9.4%, χ^2^ (1, *n* = 887) = 7.80, *p* = 0.005].

The binary logistic regression model indicated that sex, study year, distress sum score, and severity of symptoms of depression (PHQ-2 sum score) were significant predictors of serious worries with respect to the current study situation during the COVID-19 pandemic (Table 4). The other two predictors – age in categories and severity of symptoms of anxiety (GAD-2) – were not significant. Results showed that male students were significantly more likely to experience serious worries with respect to the current study situation during the COVID-19 pandemic compared to females. Students in study year 2 were significantly more likely to experience serious worries with respect to the current study situation during the COVID-19 pandemic compared to all other students. In addition, higher distress was associated with a higher likelihood for reporting serious worries with respect to the current study situation during the COVID-19 pandemic. A one-point increase in the distress scale is associated with an increase of serious worries of 38.1%. Furthermore, more severe symptoms of depression (PHQ-2) were associated with a higher likelihood for reporting serious worries with respect to the current study situation during the COVID-19 pandemic. A one-point increase in the PHQ-2 scale was associated with an increase of serious worries of 35.6% (Table 4).

**TABLE 4:** The binary logistic regression model on the association of sex, study year, distress, and depression with serious worries in medical students during the COVID-19 pandemic.

	OR	CI	*p*	*f* ^2^
**Sex**				
Male	Reference		n.a.	0.205
Female	1.886	1.208 – 2.947	**0.005**	
**Study year**				
Year 1	3.170	1.638– 6.133	**0.001**	
Year 2	Reference		n.a	
Year 3	4.140	2.123–8.073	**<0.001**	
Year 4	5.315	2.790–10.126	**<0.001**	
Distress (DT)	1.381	1.223–1.559	**<0.001**	
Depression symptoms (PHQ-2)	1.356	1.148 – 1.600	**<0.001**	

*OR, odds ratio; p, p-value; f^2^, effect size Cohen’s f^2^.*

*According to Cohen’s (1988) guidelines, we considered f^2^ = 0.02 to be a small effect, f^2^ = 0.15 as a medium effect, and f^2^ = 0.35 as a large effect.*

*Bold font indicates statistical significance.*

### Qualitative Data

A total of 456 students (51.4% of all the participants, among them 309 females, 67.8%) provided optional free-text answers with respect to the question: “What comes to mind first when thinking about your current study situation?” We identified 10 categories in a multistage inductive process. Students most frequently reported concerns about missing relevant practical learning experience, difficulties with self-regulated learning and self-motivation due to the new learning environment, study-related worries, and uncertainty. Year 4 students reported significantly more worries about the lack of practical training than students from study years 1 to 3. We identified other recurring themes with respect to study-related concerns during the initial phase of the COVID-19 pandemic. The results according to the study year are shown in Table 5.

**TABLE 5:** Categories, examples, and quantified responses for the question “What comes to mind first when thinking about your current study situation?” for the total sample and by study year.

*“What comes to mind first when thinking about your current study situation?”*		Total	Year 1	Year 2	Year 3	Year 4			
Category and subcategory	Example	(*n* = 887)	(*n* = 307)	(*n* = 192)	(*n* = 210)	(*n* = 78)	*X* ^2^	df	*p*
		*n* (%) mentioned	*n* (%) mentioned	*n* (%) mentioned	*n* (%) mentioned	*n* (%) mentioned			
Lack of practical training, i.e., bedside teaching, laboratory sessions • Concerns to miss out on relevant practical learning experience • Impedes deeper understanding and application of knowledge	“I think that I am missing out on important learning content. Bedside teaching and contact with patients and proper exchange with lecturers cannot be replaced by textbook and PowerPoint presentations. I am worried that I will miss this knowledge both as a future doctor and in the exam.” “The lack of contact with patients. Through clinical practical application, newly learned clinical pictures could be better understood and learned in greater depth.”	128 (14.4)	27 (8.8)	32 (16.7)	35 (16.7)	34 (19.1)	12.67	3	**0.005**
Difficulties with self-regulated learning and self-motivation • Self-motivation • Difficulties with self-regulated learning	“I find it increasingly difficult to motivate myself for the monotonous work at home alone at the laptop and my satisfaction with the “work done” is very low.” “The fact that I have done absolutely nothing for university yet and the first module is already over.”	125 (14.1)	40 (13.0)	25 (13.0)	30 (14.3)	30 (16.9)	1.60	3	0.660
Study-related worries and uncertainty • Study-related uncertainty • Worries regarding clinical internship year	“Uncertainty of the further course of studies and exam participation.” “I realize that I am losing interest in my studies. In addition, I am worried about the extent to which I will have to bear professional losses when it comes to STEX [Second Part of the Medical Examination] and PJ [final clinical year].”	121 (13.6)	39 (12.7)	28 (14.6)	26 (12.4)	28 (15.7)	1.32	3	0.725
Lack of interaction with faculty and peers • Learner-to-faculty (i.e., feedback, clarity of expectations) • Peer-to-peer (i.e., cooperation, support)	“There is a lack of feedback, which is particularly important in bedside teaching. There, you first learn how to apply the theory in a meaningful way in everyday clinical practice, and gaps in knowledge/understanding are quickly noticed and can be eliminated directly or afterward. At the moment, I don’t know which associations are actually important in the clinic, and how individual findings are evaluated in the interaction (case studies/bedside teaching help a lot here).” “No contact with fellow students. The interactive exchange between the students is missing. Even to hear that one or the other has a problem there, or just “quickly” explains something.”	72 (8.1)	26 (8.5)	20 (10.4)	16 (7.6)	10 (5.6)	2.97	3	0.396
Worries regarding financing, health, uncertainty and distress	“I belong to the risk group and wonder how to do the multiple choice exam without taking a risk.”	55 (6.2)	17 (5.5)	16 (8.3)	11 (5.2)	11 (6.2)	2.07	3	0.558
Social isolation	“The lack of personal contact, even in private, and related to this, (especially at the beginning of the pandemic) not really knowing what to do with yourself.”	52 (5.9)	19 (6.2)	13 (6.8)	14 (6.7)	6 (3.4)	2.60	3	0.458
Postponed exams and clerkships	“It also threw me off track that ENF [Examination Normal Function, for details see Rheingans et al. (2019)] was canceled, which I had been working toward for months with quite a lot of pressure to perform. Then, to be slowed down so shortly before the finish line threw me off track for a few weeks after the cancelation of ENF. I would have liked some support from the dean’s office. To be honest, the thought of having to take this exam again in the summer makes my stomach hurt.”	51 (5.7)	3 (1.0)	33 (17.2)	5 (2.9)	9 (5.1)	62.65	3	** < 0.001**
Changed learning environment	“I found it increasingly difficult to study in this module, as I spend most of my time in my dorm, which is not necessarily quiet //Normally I study in the library or seminar rooms, this was unfortunately not possible now.”	45 (5.1)	16 (5.2)	10 (5.2)	9 (4.3)	10 (5.6)	0.40	3	0.940
Dissatisfaction with organization, communication and nurturance by faculty	“Overall little or very late info from the dean’s office; it would also have been reassuring if there had at least been an email saying “we know there’s problem X, we’re on it.” would have come.”	45 (5.1)	6 (2.0)	16 (8.3)	8 (3.8)	15 (8.4)	15.29	3	**0.002**
Other	“At the moment, I am most burdened by having to do justice to my various tasks in life. I already have 3 children and through Covid-19 both the care and my social network broke away overnight. Full-time studies, home-schooling, kindergarten child, meal planning/cooking are many tasks that can’t be done in 24 h…”	68 (7.7)	18 (5.9)	15 (7.8)	19 (9.0)	16 (9.0)	2.42	3	0.490

*n, frequencies; X^2^, chi-squared; p, p-value.*

*Bold font indicates statistical significance.*

Furthermore, students were asked what they experienced as particularly helpful during the COVID-19 pandemic. Overall, 400 students (45.1% of all the participants, among them 289 female students, 72.6%) provided optional free-text responses. We extracted four major themes: The two most frequent aspects that were mentioned as helpful were flexibility due to digital courses and contact with family and friends. Analysis showed no significant differences of the responses between students from different study years (Table 6).

**TABLE 6:** Categories, examples, and quantified responses for the question “What comes to mind as particularly helpful in your current situation?” for the total sample and by study year.

*What comes to mind as particularly helpful in your current situation?*		Total	Year 1	Year 2	Year 3	Year 4	*X* ^2^	df	*p*
Category and subcategory	Example	(*n* = 887)	(*n* = 307)	(*n* = 192)	(*n* = 210)	(*n* = 178)			
		*n* (%) mentioned	*n* (%) mentioned	*n* (%) mentioned	*n* (%) mentioned	*n* (%) mentioned			
Flexibility due to digital courses • Self-directed learning • Individualized learning	“You can decide for yourself where and when to study, work or go to sports.” “The possibility to organize yourself. And, for example, to save lectures and then listen to them again.”	211 (23.8)	64 (20.8)	58 (30.2)	44 (21.0)	45 (25.3)	6.98	3	0.073
Contact with friends and family	“As much social interaction as possible, spending time with family, creating a regular daily routine…”	151 (17.0)	44 (14.3)	37 (19.3)	37 (17.6)	33 (18.5)	2.60	3	0.457
Balance through sports, leisure and nature-based activities	“Especially helpful for me is currently sports activities and activities in nature.”	82 (9.2)	22 (7.2)	20 (10.4)	25 (11.9)	15 (8.4)	3.80	3	0.283
Other	“To make a schedule. It would also be helpful to create groups where you can meet via Skype to work and discuss at the same time. But I personally have not done that.”	64 (7.2)	15 (4.9)	11 (5.7)	22 (10.5)	16 (9.0)	7.29	3	0.063

*n, frequencies; X^2^, chi squared; p, p-value.*

Analysis of gender differences showed that perceptions of the female students were different from perceptions of the male students in four of ten identified themes with respect to current occupation and all the themes with respect to helpful strategies (Table 7). Female students reported more frequently diverse worries and more frequently concerns with respect to postponed examinations. At the same time, female students mentioned more frequently helpful strategies with all topics than male students.

**TABLE 7:** Quantified responses for the open-ended questions by sex.

*What comes to mind first when thinking about your current study situation?*	Male students	Female students	*X* ^2^	df	*p*
	*n* = 325	*n* = 562			
Category	*n* (%) mentioned	*n* (%) mentioned			
Lack of practical training, i.e., bedside teaching, laboratory sessions	40 (12.3)	88 (15.7)	1.87	1	0.171
Difficulties with self-regulated learning and self-motivation	52 (16.0)	73 (13.0)	1.54	1	0.214
Study-related worries and uncertainty	34 (10.5)	87 (15.5)	4.40	1	**0.036**
Lack of interaction with faculty and peers	26 (8.0)	46 (8.2)	0.009	1	0.923
Worries regarding financing, health, uncertainty and distress	12 (3.7)	43 (7.7)	5.55	1	**0.018**
Changed learning environment	18 (5.5)	27 (4.8)	0.23	1	0.631
Social isolation	16 (4.9)	36 (6.4)	0.82	1	0.365
Postponed exams and clerkships	8 (2.5)	43 (7.7)	10.23	1	**0.001**
Dissatisfaction with organization, communication and nurturance by faculty	18 (5.5)	27 (4.8)	0.23	1	0.631
Other	15 (4.6)	53 (9.4)	6.75	1	**0.009**

***What comes to mind as particularly helpful in your current situation?***	**Male students**	**Female students**	***X* ^2^**	**df**	***p***
	***n* = 325**	***n* = 562**			
**Category**	***n* (%) mentioned**	***n* (%) mentioned**			

Flexibility due to digital courses	60 (18.5)	151 (26.9)	8.027	1	**0.005**
Contact with friends and family	37 (11.4)	114 (20.3)	11.55	1	**0.001**
Balance through sports, leisure and nature-based activities	16 (4.9)	66 (11.7)	11.42	1	**0.001**
Other	16 (4.9)	48 (8.5)	4.03	1	**0.045**

*n, frequencies; X^2^, chi-squared; p, p-value.*

*Bold font indicates statistical significance.*

## Discussion

In this study, we investigated mental health outcomes among medical students during the initial phase of the COVID-19 pandemic and perceptions of the students on how the learning environment had changed in a large sample of undergraduate medical students in Germany. Overall, our findings suggest that medical students experienced significant levels of distress and mental burden during the COVID-19 pandemic.

A previous study conducted at the same medical school with the same measures served as a valid context to frame our findings (Heinen et al., 2017). Comparing our results to the findings of Heinen et al. (2017), the substantial decline in all mental health measures could be attributed to the impact of the COVID-19 pandemic. Consistent with earlier findings of other studies, we found significantly higher levels of anxiety and depression among medical students compared to the German norm population (Löwe et al., 2010; Stormon et al., 2019). First year students reported the highest levels of mental burden according to the DT and PHQ-4. Previous studies found that people in their early 20s—the most common age group in students—are particularly burdened during the COVID-19 pandemic (Brooks et al., 2020; Pieh et al., 2020; Qiu et al., 2020; Dale et al., 2021).

In contrast to other studies during the COVID-19 pandemic (Lai et al., 2020; Park et al., 2020; Pieh et al., 2020; Qiu et al., 2020) and a general tendency in psychiatry research (Riecher-Rössler, 2017), we found that male students reported more frequently higher levels of depressive symptoms according to the PHQ-2 than female students. Additionally, male students were at higher risk for experiencing serious worries with respect to the study situation during the COVID-19 pandemic than females. Our qualitative data may serve as a valid context to frame these novel findings. Overall, the qualitative data indicated that worries about the lack of practical training, difficulties with self-regulated learning, study-related uncertainty, and the changed learning environment increased as the semester progressed. In this study, female students stated more frequently worries with respect to their undergraduate courses, health, financing, and general uncertainty as expected by the literature.

At first sight, it seems to be a contradiction in this study that depression was higher among males and serious worries were reported more often by male than by female students. On the other hand, with regard to qualitative data, females expressed more worries than males. It should be taken into account that completing open-ended response options require a greater amount of time and mental effort than most close-ended questions (Dillman, 2007). Thus, only 51.4 and 45.1% of all the respondents completed the two items with open-ended response options. Furthermore, the proportion of women who completed the two items with open-ended response options was higher in both the items (67.8 and 72.6%) compared to the proportion of women in the entire survey (63.4%).

Interestingly, females mentioned helpful strategies during the COVID-19 pandemic more often than male students. This might indicate the use of more efficient coping strategies during the initial phase of the COVID-19 pandemic compared to male students. Recent research showed that female students often relied on denser social networks even during the social distancing phase (Elmer et al., 2020). This strategy could have buffered the negative effects in terms of a decline in mental health among female students.

The decrease in study motivation was highest in year 2 students. These findings complement earlier empirical research indicating that undergraduate students adopted a different learning approach and a sharp decrease in intrinsic motives with the entry to clinical training (Wickramasinghe and Samarasekera, 2011; Lee et al., 2020). The curriculum structure of iMED allows the intermediate examination after the third semester. Thus, the amount of practical training increases sharply from the 4th semester, which corresponds to the second half of year 2 (Rheingans et al., 2019). The construction of the curriculum with more practical training after the 3rd semester could account for the high decrease of study motivation among 2nd year students in this study. Further, in previous research, it was discussed that motivation of male and female students differs with higher autonomous motivation among female students and higher controlled motivation among male students when compared with the opposite sex (Kusurkar et al., 2013).

Interestingly, our qualitative results mapped several scales of the Medical School Learning Environment Survey: among others, but not limited to “flexibility,” “student–student interaction,” “meaningful learning experience,” and “nurturance” (Rusticus et al., 2014). In line with recent studies, perception of the changed situation of the students included both negative and positive aspects (Elmer et al., 2020; Mohr et al., 2021). In this study, the flexibility due to digital teaching with few real-time courses is particularly noteworthy.

### Limitations

This study has some limitations that should be noted. Representativeness is limited due to data collection at a single institution. The observational cross-sectional design of this study does not allow causal statements. With 59%, the response rate may be considered as high; nevertheless, there might be a self-selection bias and particular student groups might be underrepresented. Additionally, our data included only self-reported measures. It is known that people can be biased when reporting on their own experience (Devaux and Sassi, 2016). A particular strength of this study is the consideration of quantitative and qualitative data (Frambach et al., 2013). We used well-established and valid instruments (quantitative data). With respect to the qualitative data, the conventional content analysis approach can be used when existing theories or literature is limited (Hsieh and Shannon, 2005) and offers in-depth exploration of mental health and perception of the students of their study situation during the COVID-19 pandemic. The information comes directly from the participants without predefined categories. Quantification of qualitative data can also facilitate the process of meaning discovery through pattern recognition by identifying consistencies and inconsistencies in the data, especially when analyzing large qualitative data sets (Monrouxe and Rees, 2020).

## Conclusion

The COVID-19 pandemic and its accompanying burdens and restrictions with regard to daily, occupational, and student life constitutes an unprecedented global challenge. Thus, academia and other sectors of public life cannot resort to existing concepts on how to support students in the COVID-19 pandemic circumstances. However, there is an existing body of prepandemic research on the effectiveness of interventions such as mental health programs, curricular restructuring, and mentoring programs that are associated with improved mental health among medical students (Wasson et al., 2016). In the recent statements with respect to the situation of students during the current COVID-19 pandemic, medical education researchers proposed a framework to manage student–athlete mental health during the COVID-19 pandemic including “goal setting/motivation” and “support system/social network” as potential positive influencers (Grubic et al., 2021). These aspects could be addressed among others by mentoring and mental health programs and might be valuable medical education learning environment interventions to reduce the negative impact of the COVID-19 pandemic on students. Existing interventions should be redesigned and transitioned to digital formats to provide psychological and educational support to students during the COVID-19 pandemic, as they progress through medical school. Longitudinal research is required to monitor the mental health of medical students during the COVID-19 pandemic and after.

## Data Availability Statement

Underlying datasets of the data presented in this article are not available because, ethical approval was not obtained by the ethics board to make data sharing possible outside of the listed research team.

## Ethics Statement

The local Ethics Board of the Center for Psychosocial Medicine at the University Medical Center Hamburg-Eppendorf approved the study (LPEK-0161). The participants provided their written informed consent to participate in this study.

## Author Contributions

JG designed the study, contributed to the analysis and interpretation of quantitative and qualitative data, wrote the first draft of the manuscript, and critically revised the manuscript during the internal revision process. IH contributed to the analysis and interpretation of the quantitative and qualitative data, involved in drafting the article, and critically revised the manuscript during the internal revision process. SM contributed to the acquisition and interpretation of the quantitative data, involved in drafting the article, and critically revised the manuscript during the internal revision process. CB designed the study, analyzed the quantitative data, contributed to the interpretation of the quantitative and qualitative data, and critically revised the manuscript during the internal revision process. All the authors gave their approval for the final version of the manuscript.

## Conflict of Interest

The authors declare that the research was conducted in the absence of any commercial or financial relationships that could be construed as a potential conflict of interest.

## Publisher’s Note

All claims expressed in this article are solely those of the authors and do not necessarily represent those of their affiliated organizations, or those of the publisher, the editors and the reviewers. Any product that may be evaluated in this article, or claim that may be made by its manufacturer, is not guaranteed or endorsed by the publisher.
